# Supplementary material for: Onchocerciasis and non-communicable diseases in the Bafut Health District, Cameroon: Knowledge, attitudes, and practices towards community-directed treatment with ivermectin
Source: PLoS Negl Trop Dis. 2026 Jan 2;20(1):e0013609. doi: 10.1371/journal.pntd.0013609 (PMC12782443; doi:10.1371/journal.pntd.0013609)
Supplement: S1 File — Questionnaire used to collect socio-demographic, clinical, and behavioral data from participants. (PDF) [file pntd.0013609.s001.pdf]

Questionnaire

Study Title: Onchocerciasis and Non-Communicable Diseases in the Bafut Health District, Cameroon: Knowledge, Attitudes, and Practices towards Community-Directed Treatment with Ivermectin

Section A: Sociodemographic Information

1. Gender: Male [ ] Female [ ]
2. Age (years): ________
3. Level of education: None [ ] Primary [ ] Secondary [ ] Tertiary [ ]
4. Marital status: Single [ ] Married [ ] Divorced [ ] Widowed [ ]
5. Occupation: Student [ ] Salaried worker [ ] Farmer [ ] Trader/Business person [ ] Unemployed [ ] Other (specify): __________ [ ]
6. Religion: Christian [ ] Muslim [ ] Traditional [ ] Atheist [ ] Other (specify): _________
7. Health area: __________________
8. Duration of residence in this community (years): ________

Section B: Knowledge of Onchocerciasis and CDTI

9. Have you ever heard of onchocerciasis? Yes [ ] No [ ]
10. What causes and transmits onchocerciasis? (tick all that apply)
    Filarial worm [ ] Blackfly bite [ ] Mosquito [ ] Poor personal hygiene [ ] Witchcraft [ ] Don't know [ ] Other (specify): __________
11. How are people infected with the disease? **(tick all that apply)**
    Blackfly bite [ ] Contact with infected persons [ ] Mosquito bite [ ] Sharing clothes [ ] Don't know [ ]
12. What are the signs and symptoms of onchocerciasis? **(tick all that apply)**
    Itching [ ] Swellings [ ] Skin changes [ ] Nodules [ ] Blindness [ ] Don't know [ ]
13. Is onchocerciasis preventable? Yes [ ] No [ ] I don't know [ ]
14. If yes, how can onchocerciasis be prevented? **(tick all that apply)**
    Wearing protective clothing [ ] Avoid bathing in rivers [ ] use of drugs (Ivemectin) [ ] Good personal hygiene [ ] Environmental sanitation [ ] Use of bed nets [ ]
15. Are you aware of the annual ivermectin (Mectizan) distribution? Yes [ ] No [ ]
16. How did you learn about ivermectin distribution? **(tick all that apply)**
    Community Drug Distributors (CDDs) [ ] Town Crier [ ] Radio [ ] Health worker [ ] Neighbor [ ] Other (specify): __________
17. Do you know that side effects of ivermectin are treated free of charge? Yes [ ] No [ ]

Section C: Attitudes toward Onchocerciasis and CDTI

18. Do you think onchocerciasis is a serious disease in your community? Yes [ ] No [ ] Don't know [ ]
19. Do you believe ivermectin is effective in preventing onchocerciasis? Yes [ ] No [ ] Don't know [ ]
20. Would you encourage family/friends to take ivermectin during CDTI? Yes [ ] No [ ]
21. Do you think traditional medicine can cure onchocerciasis? Yes [ ] No [ ] Don't know [ ]

Section D: Practices Related to Onchocerciasis Prevention

22. Have you ever taken ivermectin (Mectizan)? Yes [ ] No [ ]
23. When was the last time you took ivermectin?
    A few months ago [ ] Last year [ ] Last two years [ ] Never [ ]
24. Did you experience any side effects after taking ivermectin? Yes [ ] No [ ]
25. If yes, which side effects did you experience? **(tick all that apply)**
    Body swelling [ ] Itching [ ] Fever [ ] Aching joints [ ] Other (specify): __________ [ ]
26. If you ever refused ivermectin, what was your reason? **(tick all that apply)**
    Fear of side effects [ ] Did not see the need [ ] No longer had symptoms [ ] Was absent during distribution [ ] Other (specify): __________ [ ]

Section E: Onchocerciasis Symptoms and Non-Communicable Diseases

27. Do you currently experience symptoms of onchocerciasis? Yes [ ] No [ ]
28. If yes, which symptoms? **(tick all that apply)**
    Itchy skin/eyes [ ] Nodules [ ] Swellings [ ] Blurred vision [ ]
29. What time of the day do you mostly experience symptoms?
    Morning [ ] Afternoon [ ] Night [ ]
30. Have you ever been diagnosed with any non-communicable disease? Yes [ ] No [ ]
31. If yes, which of the following non-communicable diseases have you been diagnosed with? **(tick all that apply)**
    Stroke [ ] Diabetes [ ] Hypertension [ ] Arthritis/joint pain s [ ] Epilepsy [ ] None [ ]
32. Do you have a family history of any non-communicable disease? Yes [ ] No [ ]
33. If yes, which of the following non-communicable diseases have been recorded in your family? **(tick all that apply)**
    Stroke [ ] Diabetes [ ] Hypertension [ ] Arthritis [ ] Epilepsy [ ] Blindness [ ] None [ ]
