# Supplementary material for: Onchocerciasis and non-communicable diseases in the Bafut Health District, Cameroon: Knowledge, attitudes, and practices towards community-directed treatment with ivermectin
Source: PLoS Negl Trop Dis. 2026 Jan 2;20(1):e0013609. doi: 10.1371/journal.pntd.0013609 (PMC12782443; doi:10.1371/journal.pntd.0013609)
Supplement: Attachment — Submitted filename: Response to Reviewers.doc [file pntd.0013609.s004.doc]

**Reviewer #1:**

1. General comments: The study has contributed to KAP regarding onchocerciasis as well as the prevalence of NCDs. However, the authors have not clearly explained how the co-existence of onchocerciasis with NCDs will affect elimination efforts.

*We agree and have revised the Introduction (line 89-99) and Discussion (line 503-520) to clearly explain how comorbidity with non-communicable diseases may hinder onchocerciasis elimination. Specifically, we highlight how chronic conditions can reduce adherence to CDTI through fear of adverse effects, symptom overlap, and increased clinical burden, thereby lowering effective treatment coverage. These programmatic implications are now explicitly discussed and supported with appropriate references.*

2. Why were only adults recruited in the study? What sampling technique was used in selecting the adults in the household?

*Adults (≥18 years) were recruited because they are the primary decision-makers regarding ivermectin uptake within households, are able to provide informed consent, and can more reliably report prior diagnoses of non-communicable diseases. To avoid clustering and over-representation within households, only one eligible adult respondent was selected per household using a simple random selection approach among available eligible adults (line 152-158)*

3. The results revealed that participants had adequate knowledge about onchocerciasis; where did they acquire this knowledge from? Also, how did they learn about filarial worms?

*We clarified that the primary source of information on onchocerciasis and ivermectin distribution was community drug distributors (CDDs), followed by health workers through community sensitization activities. Although general awareness was high, fewer than half of respondents correctly identified the filarial worm as the causative agent, indicating that biological understanding remains limited despite high exposure to CDTI messaging.(line 271-277)*

4. Include research that has pointed out cases of ivermectin resistance in Cameroon. Also, references on the endemicity of Cameroon across all ten regions should be cited.

*We have revised the manuscript to address the concern regarding ivermectin resistance and nationwide endemicity. The Discussion especially the first paragraph (line 400-410) now clearly states that onchocerciasis remains endemic across all ten regions of Cameroon and cites evidence of persistent transmission following multiple rounds of community-directed treatment with ivermectin. Importantly, we carefully framed these observations as reflecting ongoing transmission and programmatic challenges, including suboptimal coverage and systematic non-compliance, rather than confirmed genetic resistance to ivermectin. This distinction is consistent with current evidence from Cameroon and avoids overstating resistance in the absence of molecular confirmation. The revised opening paragraph of the Discussion provides this contextual clarification and is supported by appropriate references.*

5. “In your discussion, you stated that comorbid conditions increase clinical burden in endemic areas and complicate case management and elimination efforts. How is this so? Are there documented evidences from health workers regarding this claim?”

*We expanded the Discussion to clarify how comorbid chronic conditions complicate case management and elimination efforts (line 411-425). The revised text explains that overlapping symptoms between chronic illnesses and post-ivermectin reactions increase the need for clinical assessment, counselling, referral, and pharmacovigilance, thereby placing additional strain on peripheral health services. These observations are supported by published programmatic evaluations from long-standing CDTI settings in Cameroon and other endemic areas.*

6. Why do female gender have positive attitudes compared to males? Are they more infected?

*We thank the reviewer for this comment. We clarified in the Discussion that the observed association between female gender and positive attitudes toward CDTI does not indicate higher infection rates among women (line 470-474). Rather, it likely reflects gender-related differences in health-seeking behavior, caregiving roles, and engagement with community health activities, which have been shown to influence acceptance of preventive interventions in endemic settings. This clarification has been added to the Discussion (paragraph on factors associated with attitudes)*

**Reviewer #2:**

1. Please add a section on limitation of the study, such as that cross-sectional studies do not establish causality, interviewer-administered questionnaires can introduce bias, self-reporting of data, etc.

*We revised the Limitations section to explicitly acknowledge the cross-sectional design, the use of interviewer-administered questionnaires and associated recall and social desirability biases, reliance on self-reported NCD diagnoses, and the limited generalizability of the findings (line 540-547)*

2. “Citation needed: NCDs share complications such as vision loss with onchocerciasis, and anecdotal reports suggest that people with conditions like arthritis often avoid ivermectin due to perceived or real side effects.”

*We revised the Discussion to remove anecdotal language and clarified symptom overlap between chronic conditions and onchocerciasis using cautious, literature-supported phrasing. All statements regarding treatment avoidance and adherence are now supported by published evidence.*

3. Please elaborate on the following link, which is not clear: In Cameroon, where NCDs account for more than 30% of deaths, community health workers report that up to 40% of ivermectin recipients experience adverse effects, further undermining adherence
   *The statement containing a specific percentage estimate of ivermectin-related adverse effects has been revised.*
4. Data collection instrument: Please elaborate how questions were asked (open, closed etc) and how interviewers were trained to achieve the maximum neutrality.
   *We expanded the Methods section to describe the structure of the questionnaire, the predominance of closed-ended questions, and interviewer training procedures implemented to ensure neutrality and consistency during data collection (line 176-179)*
5. Please elaborate further, whether it is the endemicity level or maybe that in hypoendemic areas more rounds of IVM have been distributed with higher coverage and thus awareness is higher due to more active program: “Furthermore, respondents living in hypoendemic areas were more knowledgeable than those in hyper-endemic zones, echoing findings that endemicity level can shape awareness and risk perception. “

*We clarified in the Discussion that the observed differences in knowledge between hypoendemic and hyperendemic areas may reflect variations in programmatic exposure, treatment coverage, and community sensitisation intensity, rather than endemicity level alone ( line 440-443)*

6. Please a) discuss their relationship with HCW, as other countries have discussed that the need for both may not be necessary and b) unclear whether CDDs have been the interviewers and how they were trained: “Making use of community drug distributors (CDDs), who were the primary source of information in this study, may offer a practical pathway to address these gaps. “

*We clarified in the Methods that community drug distributors were not involved in data collection, which was conducted by trained research assistants independent of CDTI activities (line 193-198). We also revised the Discussion to clarify the complementary roles of community drug distributors and formal health care workers in CDTI implementation, particularly for counselling, referral, and management of adverse events (line 477-452).*

7. Please elaborate, as one cause of itching could be the biting of simulia, the other one is the itching induced my mf: “The observation that many symptoms occurred in the morning and afternoon is consistent with the biting activity of Simulium blackflies, which are most active during daylight hours along fast-flowing river basins “

*We revised the Discussion to clarify that itching reported by participants may result from both Simulium blackfly biting and microfilarial-induced inflammatory responses. The revised text explicitly acknowledges these overlapping mechanisms and their implications for symptom interpretation in endemic settings (line 494-501).*

8. Please correct, as no causal link can be made in any of the points addressed in this study (albeit the OAE has been found to be associated with onchocerciasis): “While the direct causal links between onchocerciasis and most NCDs remain unclear, recent evidence points to the neurological consequences of chronic infection.

*We revised the Discussion to explicitly clarify that causal relationships between onchocerciasis and non-communicable diseases cannot be inferred from this cross-sectional study, with the exception of the well-documented association between O. volvulus infection and onchocerciasis-associated epilepsy. This clarification has been incorporated into the Discussion (line 507-513, line 593-594)..*
